# Maltose-Binding Protein Enhances Secretion of Recombinant Human Granzyme B Accompanied by *In Vivo* Processing of a Precursor MBP Fusion Protein

**DOI:** 10.1371/journal.pone.0014404

**Published:** 2010-12-22

**Authors:** Benjamin Dälken, Robert A. Jabulowsky, Pranav Oberoi, Itai Benhar, Winfried S. Wels

**Affiliations:** 1 Chemotherapeutisches Forschungsinstitut Georg-Speyer-Haus, Frankfurt am Main, Germany; 2 Department of Molecular Microbiology and Biotechnology, The George S. Wise Faculty of Life Sciences, Tel-Aviv University, Ramat Aviv, Israel; Instituto Butantan, Brazil

## Abstract

**Background:**

The apoptosis-inducing serine protease granzyme B (GrB) is an important factor contributing to lysis of target cells by cytotoxic lymphocytes. Expression of enzymatically active GrB in recombinant form is a prerequisite for functional analysis and application of GrB for therapeutic purposes.

**Methods and Findings:**

We investigated the influence of bacterial maltose-binding protein (MBP) fused to GrB via a synthetic furin recognition motif on the expression of the MBP fusion protein also containing an N-terminal α-factor signal peptide in the yeast *Pichia pastoris*. MBP markedly enhanced the amount of GrB secreted into culture supernatant, which was not the case when GrB was fused to GST. MBP-GrB fusion protein was cleaved during secretion by an endogenous furin-like proteolytic activity *in vivo*, liberating enzymatically active GrB without the need of subsequent *in vitro* processing. Similar results were obtained upon expression of a recombinant fragment of the ErbB2/HER2 receptor protein or GST as MBP fusions.

**Conclusions:**

Our results demonstrate that combination of MBP as a solubility enhancer with specific *in vivo* cleavage augments secretion of processed and functionally active proteins from yeast. This strategy may be generally applicable to improve folding and increase yields of recombinant proteins.

## Introduction

Many mammalian proteins can be expressed at high levels in the prokaryotic host *E. coli*, but frequently accumulate in a misfolded and biologically inactive form in inclusion bodies [1]. Protein solubility can either be enhanced by modifying the production process, or by protein engineering. Most efforts have been directed to chemical denaturation of purified inclusion body proteins followed by refolding procedures. However, this strategy needs to be adapted to a particular protein species, and the yields of soluble protein can vary considerably. Attempts to prevent inclusion body formation and directly increase the solubility of recombinant proteins include expression of the protein of interest fused to a heterologous protein domain as a solubilizing agent [2]. Protein domains employed in this manner comprise glutathione-S-transferase (GST) from *Schistosoma japonicum* [3], thioredoxin [4], ubiquitin [5], protein A [6], DsbA [7], domain 1 of the translation initiation factor IF2 [8], and the maltose-binding protein (MBP) of *E. coli* [9], [10].

MBP is part of a large class of proteins that aid in the uptake of small molecules [11]. While it naturally resides in the periplasm, MBP can also be expressed at high yields in the cytoplasm. For different proteins increased solubility, enhanced stability and markedly improved yields have been reported after fusion to MBP [12], [13], [14], [15]. This has been explained by the ability of MBP to act as a chaperone in the context of a fusion protein, and promote the proper folding of the fusion partner [10], [16], [17]. Here we investigated the potential of MBP to improve as part of a fusion protein the expression of human granzyme B (GrB) as a secreted recombinant protein in the methylotrophic yeast *Pichia pastoris*.

The serine protease GrB is normally released by cytotoxic lymphocytes. It enters target cells together with the pore-forming protein perforin and induces apoptosis by activating caspase-dependent and caspase-independent programmed cell death pathways [18], [19]. The availability of GrB in recombinant form is an important prerequisite for functional analysis, and is essential for application of GrB as a therapeutic effector protein [20], [21]. *P. pastoris* has previously been employed to generate recombinant GrB from different mammalian species [22], [23], [24]. This yeast represents a widely established eukaryotic expression system for proteins that are secreted to the extracellular space [25]. Fusion of the yeast mating type α-factor signal peptide to the protein of interest thereby directs it to the secretory pathway, where it becomes glycosylated before release into the culture supernatant. GrB derivatives have also been expressed as GST fusion proteins in *E. coli* [21]. Thereby proteolytic processing of such fusion proteins at a cleavage site introduced between GrB and GST domains was required during purification to separate the proteins and yield active enzyme.

To circumvent the need for *in vitro* processing, we included a synthetic furin recognition motif between the protein domains of MBP-GrB fusion proteins and investigated potential *in vivo* cleavage by endogenous furin-like proteases from yeast. Analysis of *P. pastoris* culture supernatants revealed that fusion to MBP markedly enhanced the amount of soluble GrB, while this was not the case when GrB was fused to GST. GrB was liberated from MBP by specific *in vivo* cleavage during secretion, allowing direct isolation of the enzyme in processed form. Similar results were obtained upon expression of a recombinant fragment of the ErbB2/HER2 receptor protein or GST as MBP fusions, indicating that this approach can generally be applied to enhance secretion and yields of functionally active recombinant proteins.

## Results and Discussion

### Expression of granzyme B fusion proteins in the yeast *Pichia pastoris*

**Figure 1:**
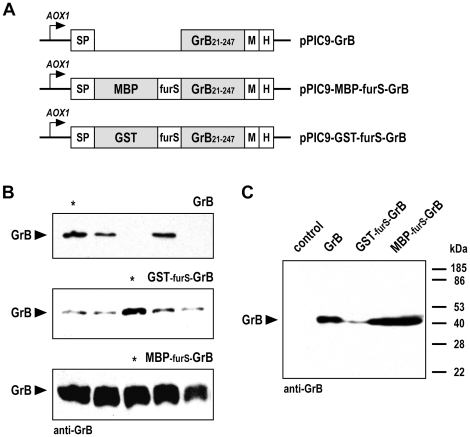
Expression of GrB in the yeast *Pichia pastoris*. (A) Constructs for expression of human granzyme B, and MBP-GrB and GST-GrB fusion proteins. *AOX1*, methanol-inducible alcohol oxidase I promoter; SP, α-factor signal peptide; furS, furin recognition motif; M, Myc tag; H, polyhistidine tag. (B) Culture supernatants of yeast clones carrying pPIC9-GrB, pPIC9-GST-furS-GrB, or pPIC9-MBP-furS-GrB were collected after induction with methanol for 3 days, and expression of GrB was analyzed by immunoblotting with GrB-specific antibody. Each lane represents an individual clone. Clones marked with an asterisk were used in subsequent experiments. (C) For comparison, culture supernatants of clones carrying the different expression constructs were analyzed together in a single immunoblot experiment as indicated. Supernatant of a yeast clone carrying empty pPIC9 served as a control.

To investigate the effects of MBP and GST on the expression and secretion of human GrB in *P. pastoris*, pPIC9-based yeast expression constructs were generated. These encode under the control of the methanol-inducible *AOX1* promoter fusion proteins consisting of an N-terminal α-factor signal peptide followed by MBP or GST, and a C-terminal domain encompassing residues 21–247 of human GrB which represents the mature form of the serine protease (Fig. 1A). To facilitate possible *in vivo* processing of the fusion proteins, a synthetic furin recognition motif Arg-Ala-Arg-Tyr-Lys-Arg-Ser (furS) was included between the fusion partners and GrB. The furS motif was previously identified as an optimal furin recognition site effectively cleaved by the enzyme in *in vitro* assays employing synthetic peptide substrates [26]. For comparison the similar construct pPIC9-GrB encoding unfused mature human GrB was included [24].

After induction with methanol, culture supernatants from individual yeast cell clones were analyzed for the presence of GrB (Fig. 1B). Only 3 out of 5 pPIC9-GrB clones expressed GrB at levels above the detection limit, whereas processed mature GrB was detected in the supernatants of all pPIC9-GST-furS-GrB or pPIC9-MBP-furS-GrB clones. Thereby expression levels of GrB derived from MBP-furS-GrB appeared more homogenous among different clones than those of GrB, or GrB derived from GST-furS-GrB. For subsequent analysis a representative pPIC9-MBP-furS-GrB yeast clone and the pPIC9-GrB and pPIC9-GST-furS-GrB clones with the highest GrB expression were selected.

### Secretion of GrB into the culture medium

Supernatants of expression cultures induced with methanol for 3 days were analyzed by immunoblotting (Fig. 1C). No bands representing full-length GST-furS-GrB or MBP-furS-GrB proteins (calculated molecular weights in non-glycosylated form 56 and 71 kDa) were detected with GrB-specific antibody. For each clone only a single protein band appeared that migrated at an apparent molecular weight of 40 kDa expected for glycosylated GrB [24]. This suggests that both fusion proteins were completely processed, presumably within the furin cleavage site which can be recognized by the endogenous furin-like yeast protease kexin [27].

**Figure 2:**
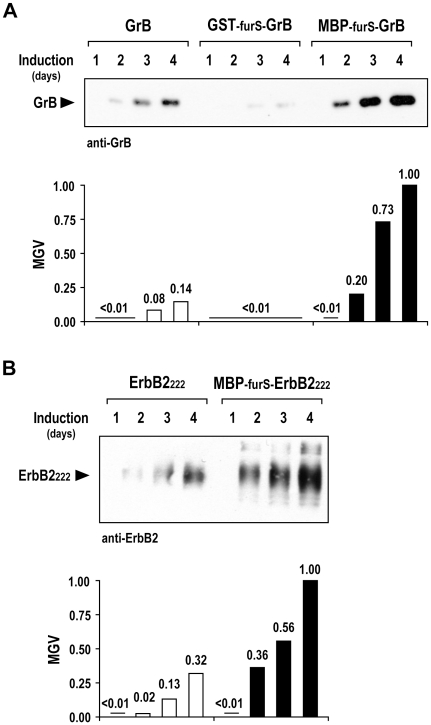
Analysis of GrB secreted into the culture medium. (A) Supernatants of yeast cultures harboring pPIC9-GrB, pPIC9-GST-furS-GrB, or pPIC9-MBP-furS-GrB were collected at the indicated time points after induction with methanol, and analyzed by immunoblotting with GrB-specific antibody. Band intensities were quantified by determining mean gray values (MGV) relative to the highest value obtained. (B) Expression of an ErbB2 protein fragment. Yeast cells carrying pPIC9-ErbB2_222_ or pPIC9-MBP-furS-ErbB2_222_ were induced with methanol, and ErbB2 protein in culture supernatants was analyzed by immunoblotting with ErbB2-specific antibody.

The highest amount of processed GrB was found in supernatant of the pPIC9-MBP-furS-GrB cell clone, with a more moderate GrB level obtained from the clone transformed with the pPIC9-GrB control construct. The lowest amount of processed GrB was detected in supernatant of the pPIC9-GST-furS-GrB cell clone (Fig. 1C). To investigate the kinetics of GrB expression, samples of culture supernatants were collected from methanol-induced cultures of pPIC9-GrB, pPIC9-GST-furS-GrB, or pPIC9-MBP-furS-GrB clones from day 1 to 4 after induction. Immunoblot analysis revealed an increasing accumulation of processed GrB over time, with the highest amounts again produced by *P. pastoris* carrying pPIC9-MBP-furS-GrB (Fig. 2A, upper panel). We found a more than 20 times higher level of processed GrB upon expression of MBP-furS-GrB in supernatant collected on day 2 when compared to unfused GrB. While there was a stronger relative increase in GrB levels in the supernatants of yeast carrying pPIC9-GrB during the following days, the GrB amount in supernatant of pPIC9-MBP-furS-GrB cells was still more than 7 times higher after induction for 4 days (Fig. 2A, lower panel). Expression of GST-furS-GrB fusion protein resulted in only low amounts of processed GrB in culture supernatants during the entire course of the experiment.

Next we investigated whether the secretion of increased levels of processed protein was dependent on the protein of interest fused to MBP-furS, using a soluble fragment of the extracellular domain of the human ErbB2/HER2 receptor protein (ErbB2_222_) as a model protein unrelated to GrB. As in the case of GrB, fusion to MBP-furS markedly enhanced the levels of the processed ErbB2_222_ protein fragment in culture supernatants during 4 days of induction (Fig. 2B), indicating that the MBP-furS domain functions as a general enhancer of the expression of secreted proteins in *P. pastoris*.

### Growth kinetics and GrB mRNA levels

**Figure 3:**
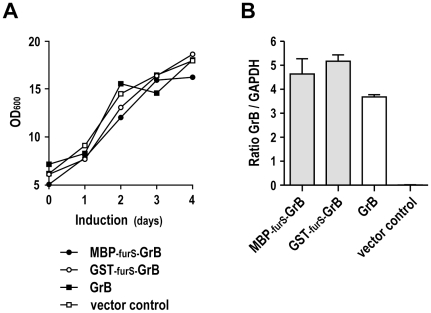
Growth kinetics of yeast clones and GrB mRNA expression. (A) Cultures of *P. pastoris* carrying pPIC9-GrB, pPIC9-GST-furS-GrB, pPIC9-MBP-furS-GrB, or empty pPIC9 vector were induced with methanol. Samples were taken every 24 h, and OD_600_ was determined as a measure for cell density. (B) Quantification of GrB mRNA levels. RNA was isolated from yeast cells carrying pPIC9-GrB, pPIC9-GST-furS-GrB, pPIC9-MBP-furS-GrB, or empty pPIC9 vector after induction with methanol for one day. cDNA was synthesized and analyzed by quantitative real-time PCR including GAPDH for normalization. Means of duplicate samples are shown. Error bars indicate SD.

During the 4 day induction *P. pastoris* carrying pPIC9-MBP-furS-GrB, pPIC9-GST-furS-GrB, pPIC9-GrB, or empty pPIC9 vector showed comparable growth rates (Fig. 3A). This demonstrates that the increased GrB levels produced by yeast cells expressing MBP-furS-GrB was not due to higher cell density or enhanced proliferation. Likewise, no differences in the growth kinetics of *P. pastoris* carrying pPIC9-ErbB2_222_ and pPIC9-MBP-furS-ErbB2_222_ were observed (data not shown).

Next we analyzed the levels of GrB and GrB fusion mRNA in yeast cells to investigate potential differences in transcription as a factor contributing to differential GrB protein expression. After induction with methanol for one day, quantitative real-time PCR revealed highest mRNA expression for GST-furS-GrB, with MBP-furS-GrB and GrB mRNA levels reaching 91% and 73% of that (Fig. 3B). These relatively small differences in mRNA expression did not reflect the observed differences in protein expression, where GST-furS-GrB resulted in the lowest levels of processed GrB. Hence, the markedly increased amount of processed GrB upon expression of MBP-furS-GrB was not due to enhanced transcription of the fusion construct. To estimate the potential effect of different codon usage within the heterologous MBP and GST domains on expression in *P. pastoris*, we calculated the codon adaptation index (CAI) for both genes. A CAI value of 1 indicates a codon choice identical to that of the host cell, while a lower CAI value indicates a bias towards less favorable codon usage [28], [29]. The calculated CAI value of 0.64 for the MBP fragment is lower than the CAI value of 0.75 for the GST fragment, making it highly unlikely that differences in codon usage account for the increased amount of GrB upon expression of MBP-furS-GrB when compared to GST-furS-GrB.

### Processing of GST and MBP fusion proteins

**Figure 4:**
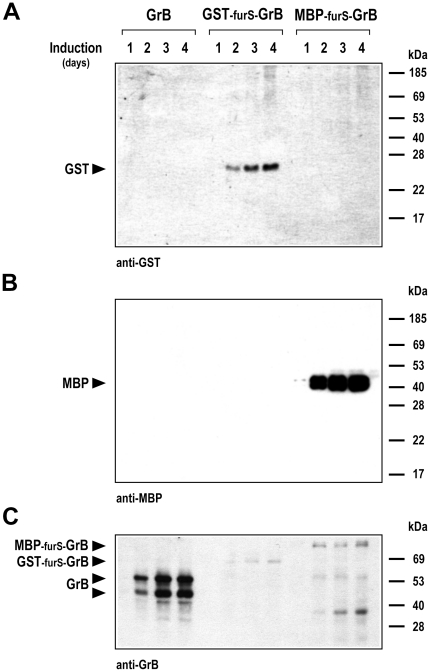
Secretion of GST and MBP into the culture medium. The presence of GST and MBP in yeast culture supernatants after induction for the indicated time periods was analyzed by immunoblotting with GST-specific (A) or MBP-specific antibodies (B). (C) Intracellular accumulation of GrB and GrB fusion proteins was investigated by analysis of cell lysates with GrB-specific antibody. The positions of the different GrB proteins are indicated by arrows. Samples analyzed were from the same cultures examined for secretion of GrB as shown in Fig. 2A.

To investigate the fate of GST and MBP domains after expression of the fusion proteins, we examined culture supernatants by immunoblot analysis with GST- and MBP-specific antibodies. Thereby no unprocessed GST-furS-GrB (Fig. 4A) or MBP-furS-GrB (Fig. 4B) proteins were detected, confirming the results with GrB-specific antibody shown above. However, we observed an increase over time of soluble GST and MBP, that reflected the previously observed levels of processed GrB (see Fig. 2A for comparison). The apparent molecular weights of the single bands detected correspond well with the calculated molecular weights of the MBP (40.6 kDa) and GST (25.6 kDa) moieties. This indicates complete processing of the fusion proteins by cleavage within the furS recognition site in the secretory pathway, resulting in secretion of processed GrB and both fusion partners GST and MBP.

Proteins that are secreted by the exocytosis pathway translocate into the membrane or lumen of the endoplasmic reticulum (ER), and are then transported to the Golgi apparatus for sorting to cellular destinations. Misfolded proteins are retained in the ER, and in some cases are retranslocated from the ER and subjected to ER-associated degradation by the ubiquitin-proteasome system [30]. To investigate potential intracellular retention of the GrB fusion proteins, yeast cell lysates were prepared and analyzed with GrB-specific antibody (Fig. 4C). For unfused GrB included for comparison we detected two bands in the intracellular fraction migrating at approximately 44 and 53 kDa, apparently corresponding to processed glycosylated GrB and glycosylated GrB still containing the signal peptide. In contrast, only marginal amounts of unprocessed GST-furS-GrB and MBP-furS-GrB fusion proteins were found, together with two additional smaller bands likely representing MBP-furS-GrB degradation products. These data show that a large proportion of unfused GrB was retained in an intracellular compartment likely representing the ER, while GST and MBP domains both promoted secretion of GrB. However, only MBP was able to enhance the overall level of GrB.

### Influence of the furin recognition motif on GrB cleavage and activity

**Figure 5:**
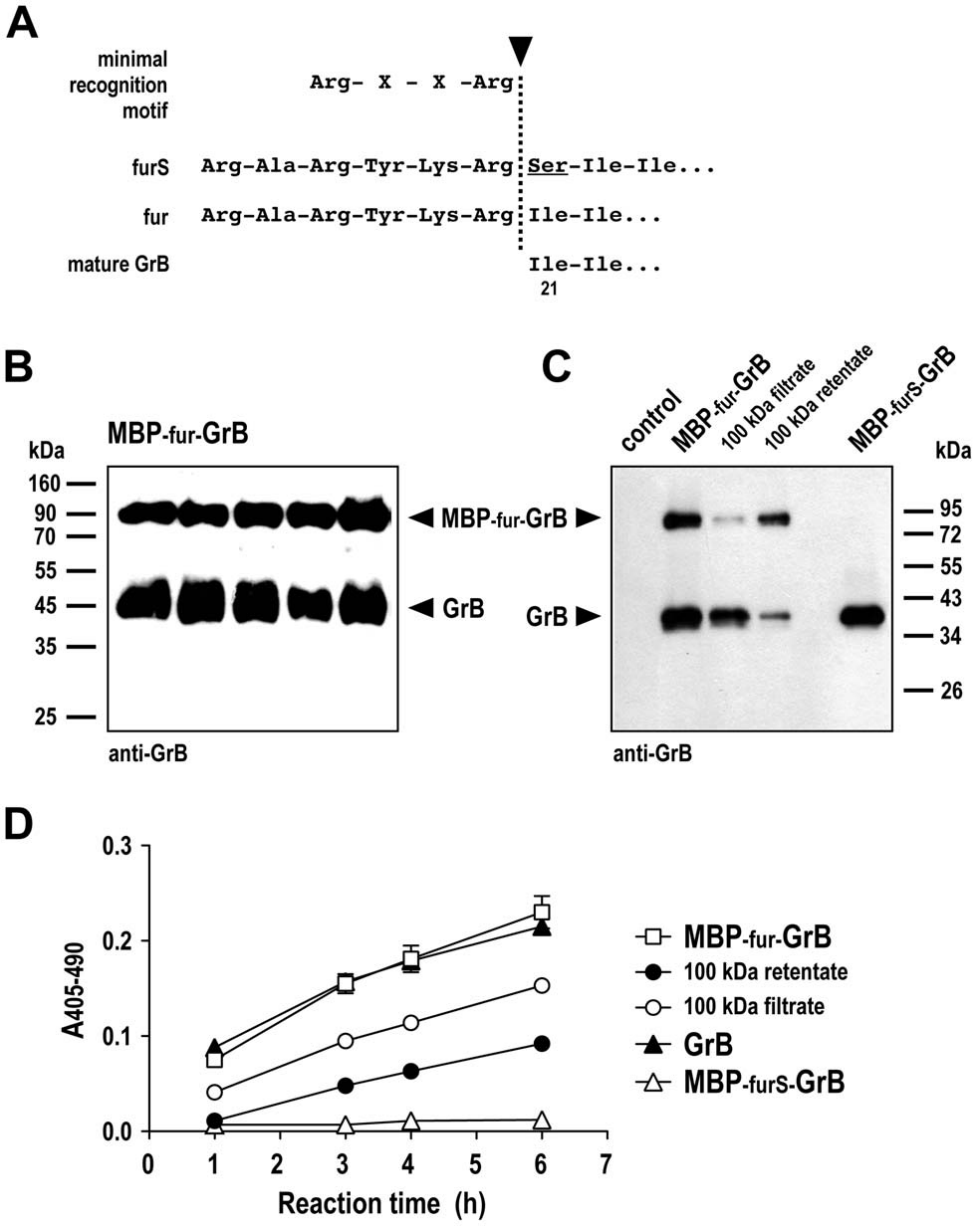
Processing of MBP-GrB fusion proteins. (A) Variants of the furin recognition motif either containing (furS) or lacking (fur) an additional C-terminal serine residue. (B) Culture supernatants of yeast clones carrying pPIC9-MBP-fur-GrB were collected after induction with methanol for 3 days, and expression and processing of the GrB fusion protein was analyzed by immunoblotting with GrB-specific antibody. (C) Samples enriched for processed (100 kDa filtrate) or unprocessed MBP-fur-GrB protein (100 kDa retentate) were prepared by filtration of culture supernatant of a representative MBP-fur-GrB clone through Amicon filters with 100 kDa membranes, and analyzed by immunoblotting with GrB-specific antibody. Initial MBP-fur-GrB containing supernatant and supernatant from an MBP-furS-GrB clone were included for comparison. Supernatant of yeast cells carrying empty pPIC9 served as a control. The lower apparent molecular weight of processed GrB in (C) when compared to (B) is most likely due to a lower degree of glycosylation (calculated molecular weight for processed GrB in non-glycosylated form: 28.4 kDa). (D) Enzymatic activity of GrB proteins from culture supernatants and kinetics of substrate cleavage were analyzed in a colorimetric peptide cleavage assay. The MBP-fur-GrB or MBP-furS-GrB containing supernatants, and the MBP-fur-GrB samples enriched for processed (100 kDa filtrate) or unprocessed protein (100 kDa retentate) analyzed in (C) were tested as indicated. A purified recombinant GrB derivative (GrB) was included as a positive control. Supernatant of yeast cells carrying empty pPIC9 displayed no GrB activity (data not shown). Means of triplicate samples are shown. Error bars indicate SEM.

GrB is naturally expressed as a pre-pro-enzyme, that requires stepwise removal of the signal peptide and an N-terminal activation dipeptide to yield active protein [31]. Hence, for expression of recombinant GrB in *P. pastoris* we previously fused the sequence of mature GrB directly to the α-factor signal peptide in pPIC9-GrB, allowing secretion of fully processed and active enzyme into the culture supernatant [24]. In contrast to the minimal furin recognition motif Arg-X-X-Arg which is cleaved after the C-terminal arginine residue, the optimized furS cleavage site contains an additional C-terminal serine residue which is present in many physiological substrates of furin [26], and resides at the N-terminus of the cleavage product after processing (Fig. 5A). To enable production of GrB with an N-terminus identical to that of the natural protein, we generated a modified pPIC9-MBP-fur-GrB construct which carries a furin recognition motif (fur) lacking the C-terminal serine of furS.

After induction with methanol we detected significant levels of unprocessed full-length MBP-fur-GrB in supernatants of pPIC9-MBP-fur-GrB clones in addition to the major band corresponding to processed GrB (Fig. 5B). This is in contrast to the findings after expression of MBP-furS-GrB, and indicates that furS represents a more optimal recognition site than fur also for furin-like proteases in yeast. To enrich the processed GrB fragment for subsequent analysis, MBP-fur-GrB containing culture supernatant was filtered through membranes with a 100 kDa molecular weight cut-off, yielding a filtrate with a high proportion of *in vivo* processed GrB and a retentate enriched for unprocessed MBP-fur-GrB (Fig. 5C). Catalytic GrB activity in these samples was then analyzed in a peptide cleavage assay using the synthetic GrB substrate Ac-IETD-pNA [24]. As expected, protein from yeast cells expressing MBP-fur-GrB readily cleaved the GrB-specific substrate with kinetics similar to those of a purified recombinant GrB derivative included for comparison, demonstrating the presence of enzymatically active GrB (Fig. 5D). Thereby samples enriched for unprocessed MBP-fur-GrB showed lower enzymatic activity than samples containing a larger proportion of processed GrB, indicating that processing is required to yield the active form. In contrast, no GrB activity was detected in supernatant of MBP-furS-GrB expressing cells despite complete *in vivo* processing and liberation of the GrB domain. This strongly suggests that after cleavage of MBP-furS-GrB the C-terminal serine of the furS motif was retained at the N-terminus of GrB, accounting for the lack of enzymatic activity [24].

### Expression, processing and enzymatic activity of MBP-GST fusion proteins

**Figure 6:**
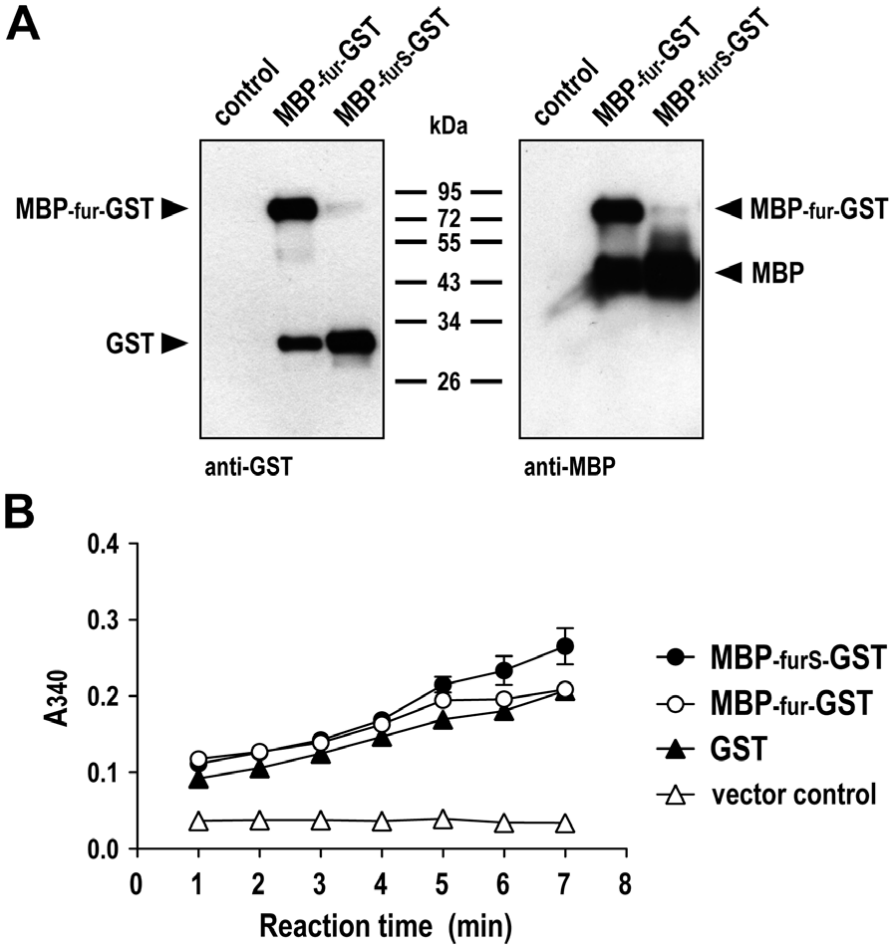
Processing and enzymatic activity of MBP-GST fusion proteins. (A) Culture supernatants of representative yeast clones carrying pPIC9-MBP-fur-GST or pPIC9-MBP-furS-GST were collected after induction with methanol for 3 days, and expression and processing of the MBP-GST fusion proteins was analyzed by immunoblotting with GST-specific (left panel) or MBP-specific antibody (right panel). Supernatant of yeast cells carrying empty pPIC9 served as a control. (B) Enzymatic activity of MBP-GST fusion proteins and kinetics of conjugation of glutathione to synthetic CDNB substrate was analyzed in a colorimetric assay. The MBP-fur-GST or MBP-furS-GST containing supernatants analyzed in (A) were tested as indicated. Commercially available recombinant GST was included as a positive control. Supernatant of yeast cells carrying empty pPIC9 served as negative control. Means of triplicate samples are shown. Error bars indicate SEM.

To investigate the influence of MBP on the expression and catalytic activity of an enzyme unrelated to GrB, we fused GST C-terminally to the MBP-fur or MBP-furS domains. Similar to the corresponding GrB fusions, the resulting MBP-fur-GST and MBP-furS-GST proteins were highly expressed in *P. pastoris*, and readily secreted into the culture supernatant (Fig. 6A). This was in marked contrast to the poor expression of the GST-furS-GrB fusion protein carrying the GST domain at the N-terminus and lacking MBP (see Fig. 1 for comparison), confirming that the differential expression of GST and MBP, if not fused to each other, is not due to differences in codon usage. Like in the case of MBP-GrB fusion proteins, MBP-fur-GST was only partially processed by *in vivo* cleavage, whereas almost quantitative processing was observed for MBP-furS-GST, liberating MBP and GST domains (Fig. 6A).

Enzymatic GST activity in yeast culture supernatants was analyzed by measuring conjugation of glutathione to a synthetic substrate. Thereby very similar activities were found for MBP-fur-GST and MBP-furS-GST preparations irrespective of the level of cleavage within their fur or furS sites. These results demonstrate that unprocessed MBP-fur-GST fusion protein, and GST generated by *in vivo* processing of MBP-fur-GST or MBP-furS-GST are enzymatically active to a similar degree. Accordingly, in contrast to GrB, in the case of GST the N-terminal serine residue left upon cleavage of furS does not affect functionality of the enzyme.

### Conclusions

Expression of recombinant proteins in the yeast *P. pastoris* represents an attractive alternative to prokaryotic expression systems [25]. Proteins can be efficiently secreted into the culture supernatant, which aids proper folding and allows posttranslational modification such as glycosylation [24], [32], [33], [34]. Nevertheless, protein expression levels comparable to those of bacteria can often not be achieved. Here we demonstrated that expression of human GrB in *P. pastoris* was markedly enhanced upon fusion to bacterial MBP as a solubilizing agent, while this was not the case after fusion to GST. Unlike unfused GrB, which was retained to a large extent in the intracellular compartment suggesting incorrect folding, GrB linked to MBP was efficiently secreted. Similar results were obtained upon expression of a fragment of the ErbB2/HER2 receptor protein or GST as MBP-furS fusions. This indicates that also in yeast the MBP domain acts like a chaperone and promotes proper folding of the fusion partner [10], [16], [17]. As in the case of GrB, fusion to a solubilizing protein domain may interfere with the biological activity of the protein of interest, requiring separation of the protein domains after expression. This is usually achieved by *in vitro* processing with the help of an exogenously added protease that cleaves a site introduced between the fusion partners.

To circumvent the necessity of such extensive *in vitro* manipulation, we separated the MBP domain from GrB by a furin recognition site to allow cleavage of the fusion protein *in vivo*. Indeed, inclusion of the optimized furS motif resulted in quantitative processing of MBP fusion proteins in the secretory pathway, demonstrating that this site is efficiently recognized by a furin-like yeast protease such as kexin [27]. Comparative analysis of GrB enzymatic activity as a sensitive readout for correct processing of the protein's N-terminus indicated that cleavage of furS and fur recognition sites apparently occurred at the expected positions. The terminal serine of furS is thereby left at the N-terminus of processed GrB, rendering the enzyme inactive. In the case of GrB a more basic furin recognition site must therefore be employed to generate active enzyme with its natural N-terminus. Nevertheless, also for MBP-fur-GrB the general expression-enhancing effect of the MBP fusion partner was retained. Albeit less complete than in the case of furS, *in vivo* cleavage of MBP-fur-GrB was sufficiently efficient to yield overall levels of processed GrB comparable to those generated by expression of MBP-furS-GrB.

Recently, Li and colleagues independently reported enhanced secretion of proteins of interest from *P. pastoris* after fusion to MBP [35]. In their study processing between MBP and cargo protein was found irrespective of the presence of a Factor Xa or an enterokinase cleavage site within the linker. This was attributed to possible recognition of an as yet undefined three-dimensional structure in MBP by an unknown *P. pastoris* protease, followed by cleavage within the linker region or the cargo protein in a quasi random fashion [35]. In contrast, in our case the efficiency of *in vivo* cleavage of MBP-GrB and MBP-GST fusion proteins was strictly dependent on the type of cleavage site fur or furS, which only differ in one amino acid residue. Furthermore, the high enzymatic activity observed for GrB generated upon fur cleavage and the complete lack of enzymatic activity of GrB generated upon processing of the furS site indicate that cleavage of the MBP fusion proteins did not occur randomly but at the predicted positions.

While the serine residue left after cleavage of the more efficient furS site prevented GrB activity, it did not interfere with the functionality of GST, which like many other enzymes does not require exact N-terminal trimming to yield the enzymatic activity of the wildtype protein. Our results indicate that this novel combination of a solubilizing protein domain fused to the protein of interest via a cleavage site for *in vivo* processing can be applied as a general strategy to improve the yields of functionally active proteins. A similar approach may also be followed for mammalian cells, where furin is present in the secretory pathway, and even prokaryotic expression systems, where bacterial subtilisins share the substrate specificity of furin [36].

## Materials and Methods

### Construction of yeast expression plasmids

DNA fragments encoding MBP and GST were generated by PCR using plasmids pMALc-NN-EGFP (MBP) [16] or pGEX-4T-1 (GST) (GE Healthcare, Freiburg, Germany) as templates and oligonucleotide primers that introduce 5′ *Nde*I and 3′ *Xba*I restriction sites. cDNA fragments that encode mature human granzyme B (GrB; amino acid residues 21–247) preceded by sequences encoding the furin recognition motifs Arg-Ala-Arg-Tyr-Lys-Arg-Ser (furS) or Arg-Ala-Arg-Tyr-Lys-Arg (fur) were generated by PCR using plasmid pPIC9-GrB as a template [24] and oligonucleotide primers that introduce 5′ *Xba*I and 3′ *Xho*I restriction sites. The PCR products were assembled stepwise as *Nde*I/*Xba*I and *Xba*I/*Xho*I fragments in the pBluescript KS^+^ derivative pBMH that contains a modified multiple cloning site and sequences encoding Myc (M) and polyhistidine (H) tags [32]. From the resulting plasmids DNA fragments encoding MBP-furS-GrB, MBP-fur-GrB, and GST-furS-GrB fusions that also harbor C-terminal Myc and polyhistidine tags were derived by digestion with *Avr*II and *Not*I, and inserted into the respective restriction sites of plasmid pPIC9 (Invitrogen, Darmstadt, Germany), resulting in the yeast expression vectors pPIC9-MBP-furS-GrB, pPIC9-MBP-fur-GrB, and pPIC9-GST-furS-GrB (Fig. 1A). In a similar fashion the yeast expression vectors pPIC9-ErbB2_222_ and pPIC9-MBP-furS-ErbB2_222_ for expression of an N-terminal fragment of the human ErbB2 protein (amino acid residues 1-222, ErbB2_222_) and an MBP-furS-ErbB2_222_ fusion were assembled using an ErbB2_222_ cDNA fragment derived from plasmid pSW5-ErbB2_222_ [37]. The expression vectors pPIC9-MBP-fur-GST and pPIC9-MBP-furS-GST for expression of MBP-fur-GST and MBP-furS-GST fusions were derived by exchanging the fur-GrB fragment in pPIC9-MBP-fur-GrB with fur-GST and furS-GST PCR fragments generated with plasmid pGEX-4T-1 as a template.

### Expression of recombinant proteins in the yeast *Pichia pastoris*

*P. pastoris* GS115 cells (Invitrogen) were transformed with pPIC9 derivatives by electroporation, and positive clones were selected according to the manufacturer's recommendations. Yeast cells carrying plasmid pPIC9-GrB [24] for expression of unmodified GrB served as control. Single colonies were grown in 10 ml buffered glycerol complex medium (pH 8) for 2 days at 28°C. For induction of protein expression, cultures were diluted to an OD_600_ of 6 in 25 ml of buffered methanol complex medium (pH 8) containing 2% methanol, and incubated for another 3 to 4 days at 28°C with addition of methanol every 24 h. Growth of yeast cultures was monitored by measuring OD_600_ every 24 h, and samples for subsequent analysis were collected at daily intervals. Yeast cells and culture supernatants from harvested samples were separated by centrifugation. For detection of recombinant proteins, culture supernatants (secreted fraction) and yeast pellets resuspended in PBS (intracellular fraction) were subjected to SDS-PAGE and immunoblot analysis with GrB-specific 2C5 (Santa Cruz), or ErbB2-specific FRP5 [38] monoclonal antibody, followed by HRP-conjugated secondary antibody and chemiluminescent detection with the ECL kit (GE Healthcare). Bands were quantified by densitometry using ImageJ 1.32j software [39] to determine mean gray values, calculated relative to the highest values obtained which were set to 1. MBP and GST fusion proteins were also analyzed by immunoblotting with MBP-specific monoclonal antibody MBP-17 (Abcam, Cambridge, UK), or polyclonal GST-specific antibody (GE Healthcare) followed by HRP-conjugated secondary antibody and chemiluminescent detection.

### Isolation of yeast RNA and quantitative real-time PCR

Yeast expression cultures were induced for one day with 2% methanol and adjusted to an OD_600_ of 8. Cell pellets from 1 ml samples of the cultures were collected by centrifugation, and resuspended in 100 µl of buffer containing 1 M sorbitol, 0.1 M EDTA pH 7.4, 0.1% mercaptoethanol, and 50 units lyticase (Sigma-Aldrich, Deisenhofen, Germany). Spheroblasts were allowed to form by incubation for 30 min at 30°C with moderate agitation, and then collected by centrifugation for 5 min at 300×g. Total RNA was isolated from spheroblasts using the RNeasy Mini Kit (Qiagen, Hilden, Germany) following the manufacturer's recommendations, and cDNA was synthesized from 1 to 5 µg of RNA following a standardized protocol as described [40]. Duplicate TaqMan PCR were performed on an Abi PRISM 7700 sequence detection system (Applied Biosystems, Weiterstadt, Germany) with standard conditions (50°C for 2 min, 95°C for 10 min, 45 cycles at 95°C for 15 sec, 60°C for 1 min) using Platinum RTS qPCR-Super-Mix-UDG with Rox (Invitrogen) according to the manufacturer's recommendations. For amplification of the GrB fragment, 2.5 µl of template were used in 25 µl reactions containing oligonucleotide primers 5′-TGGAGGCCCTCTTGTGTGTAA-3′ and 5′-GCATGCCATTGTTTCGTCC-3′, and the GrB-specific probe 5′-AAGGTGGCCCAGGGCATTGTCTC-3′ labeled with FAM™ and TAMRA™ at 5′ and 3′ ends, respectively. A yeast GAPDH fragment was amplified for normalization. All primers and probes were used at a final concentration of 200 nM. Absolute copy numbers of GrB and GAPDH mRNA were determined by simultaneously analyzing standard titrations of GrB and GAPDH plasmids.

### Analysis of codon usage

The codon adaptation index [28], [29] for the MBP and GST gene fragments was calculated as a measure for the potential influence of different codon usage on the expression of MBP and GST fusion proteins in *P. pastoris* using a free online tool accessible at www.genscript.com/cgi-bin/tools/rare_codon_analysis (GenScript, Piscataway, NJ).

### Granzyme B activity assay

Enzymatic activity of recombinant GrB was analyzed using the synthetic GrB peptide substrate Ac-IETD-pNA (Acetyl-Ile-Glu-Thr-Asp-p-Nitroanilide) (Alexis, Grünberg, Germany). Cleavage reactions were prepared in triplicates in 96 well plates in a total volume of 100 µl per well, containing 200 µM Ac-IETD-pNA (from a 20 mM stock solution in DMSO) in reaction buffer (10 mM HEPES pH 7.4, 140 mM NaCl, 2.5 mM CaCl_2_), and 20 µl of yeast culture supernatants collected after induction of MBP-fur-GrB or MBP-furS-GrB expression for 3 days. Likewise GrB activity was determined in MBP-fur-GrB samples containing equivalent protein amounts but enriched for either unprocessed (retentate) or processed MBP-fur-GrB protein (filtrate) by filtration of culture supernatants through Amicon Ultra centrifugal filters with 100 kDa membranes (Millipore, Schwalbach, Germany). A highly purified recombinant GrB derivative [20] was included as a positive control. Samples were incubated for 1 to 6 h at 37°C, and cleavage of the GrB substrate was quantified by measuring the absorbance at 405 nm and 490 nm with a microplate reader (Molecular Devices, Ismaning, Germany). Peptide substrate incubated in reaction buffer without recombinant proteins served as blank. Culture supernatant from a yeast clone transformed with empty pPIC9 vector served as negative control.

### Glutathione-S-transferase activity assay

Enzymatic activity of MBP-fur-GST and MBP-furS-GST proteins was analyzed using a GST assay kit (Sigma-Aldrich) following the manufacturer's recommendations. Yeast culture supernatants were collected after induction of protein expression for 3 days, 10×concentrated using Amicon Ultra centrifugal filters with 10 kDa membranes (Millipore), and dialyzed against PBS. Reactions were prepared in triplicates in 96 well plates in a total volume of 200 µl per well, containing 5 µl of concentrated culture supernatant, 2 mM reduced L-glutathione, and 1 mM CDNB (1-chloro-2,4-dinitrobenzene) substrate. Samples were incubated for 1 to 7 min at 25°C, and GST activity was quantified by measuring the absorbance at 340 nm with a microplate reader. Buffered substrate solution without recombinant proteins served as blank. Enzymatically active GST provided with the kit was included as a positive control. Concentrated culture supernatant from a yeast clone transformed with empty pPIC9 vector served as negative control.
